# USP29 Deubiquitinates SETD8 and Regulates DNA Damage-Induced H4K20 Monomethylation and 53BP1 Focus Formation

**DOI:** 10.3390/cells11162492

**Published:** 2022-08-11

**Authors:** Yeray Hernández-Reyes, María Cristina Paz-Cabrera, Raimundo Freire, Veronique A. J. Smits

**Affiliations:** 1 Unidad de Investigación, Hospital Universitario de Canarias, 38320 Santa Cruz de Tenerife, Spain; 2 Instituto de Tecnologías Biomédicas, Universidad de La Laguna, 38200 Santa Cruz de Tenerife, Spain; 3 Escuela de Doctorado y Estudio de Postgrado, Universidad de la Laguna, 38200 Santa Cruz de Tenerife, Spain; 4 Facultad de Ciencias de la Salud, Universidad Fernando Pessoa Canarias, 35450 Las Palmas de Gran Canaria, Spain

**Keywords:** SetD8/Set8/PR-Set7/KMT5A, USP29, 53BP1, H4K20me, DNA damage response, histone methylation, cell cycle

## Abstract

SETD8 is a histone methyltransferase that plays pivotal roles in several cellular functions, including transcriptional regulation, cell cycle progression, and genome maintenance. SETD8 regulates the recruitment of 53BP1 to sites of DNA damage by controlling histone H4K20 methylation. Moreover, SETD8 levels are tightly regulated in a cell cycle-dependent manner by ubiquitin-dependent proteasomal degradation. Here, we identified ubiquitin-specific peptidase 29, USP29, as a novel regulator of SETD8. Depletion of USP29 leads to decreased SETD8 protein levels, an effect that is independent of the cell cycle. We demonstrate that SETD8 binds to USP29 in vivo, and this interaction is dependent on the catalytic activity of USP29. Wildtype USP29 can deubiquitinate SETD8 in vivo, indicating that USP29 directly regulates SETD8 protein levels. Importantly, USP29 knockdown inhibits the irradiation-induced increase in H4K20 monomethylation, thereby preventing focus formation of 53BP1 in response to DNA damage. Lastly, depletion of USP29 increases the cellular sensitivity to irradiation. These results demonstrate that USP29 is critical for the DNA damage response and cell survival, likely by controlling protein levels of SETD8.

## 1. Introduction

Posttranslational modifications (PTMs) of histones are critical for the regulation of several cellular processes. The N-terminal tails of the four core histones are reversibly modified by phosphorylation, ubiquitination, methylation, and acetylation, and these modifications influence chromatin dynamics and epigenetic functions. Acetylation of histones usually results in changes in chromatin structure and is associated with transcriptional activation. In contrast, histone lysine (Lys, K) methylation, occurring on histone H3 and H4, can either activate or repress transcription, depending on the site of methylation [1]. Methylation of Lys9 and Lys27 of histone H3 (H3K9 and H3K27) is an indication of silenced transcription and heterochromatin structure, whereas methylation of Lys 4 or 36 (H3K4 and H3K36) is associated with transcriptional activation in the euchromatin [2]. Histone H4 Lys20 (H4K20) methylation is linked to chromatin condensation and negatively affects transcription, but also impacts cell cycle progression, DNA replication, and the DNA damage response, depending on the methylation status. Monomethylated H4K20 (H4K20me1) can be converted to H4K20me2/3, transitions that are mediated by different methyltransferases [2]. SETD8, also known as Set8, PR-Set7, or KMT5A, is a member of the SET domain family of histone lysine methyltransferases and, to date, the only known enzyme that catalyzes monomethylation of H4K20 in eukaryotes [3]. Since H4K20me2/3 depends on H4K20me1, loss of SETD8 catalytic activity affects several essential cellular processes such as gene transcription, cell cycle regulation, and DNA replication [4,5].

In addition, methylation of H4K20 by SETD8 was also shown to impact the control of the response to DNA double-strand breaks (DSBs) [6]. The regulation of the DNA damage response requires the correct and timely coordination of a multitude of signaling events, in which PTMs, both of histones and of regulatory proteins in the pathway, play a critical role, through activation and recruitment of repair proteins [7]. For example, the recruitment of 53BP1, a mediator protein critical for the DSB repair pathway choice by directing DSB repair via nonhomologous end-joining (NHEJ), depends on the methylation of H4K20 together with the RNF8-dependent degradation of competing H4K20me readers, deacetylation of H4K16, and RNF168-mediated H2AK15 ubiquitination [8,9]. Interestingly, SETD8 and H4K20me1 were shown to accumulate at sites of DNA damage [10,11,12,13]. Furthermore, the catalytic activity of SETD8 is required for the recruitment of 53BP1 to DSBs. H4K20 monomethylation by SETD8 was thought to facilitate the recruitment of the Suv4-20 methyltransferase, which catalyzes H4K20me2 and subsequent 53BP1 binding to sites of DNA lesions. In this way, SETD8 promotes DSB repair via NHEJ [10,12,13].

In addition to directing the recruitment of repair factors, PTMs, particularly ubiquitination, are also critical for controlling the levels of many proteins involved in this pathway via proteasome-dependent degradation [9,14]. SETD8 is also regulated by ubiquitin-mediated proteasomal degradation, resulting in a fluctuation of SETD8 protein levels during the cell cycle, with low levels in S phase [10,15,16,17]. In addition, SETD8 is degraded in response to DNA damage by UV light [10,18,19]. With the identification of the E3 ubiquitin ligases SCF^Skp2^, APC^Cdh1^, CRL4^Cdt2^, and SCF^βTrCP^ in such regulation of SETD8 protein levels, quite a bit is known about how SETD8 is sent for degradation [10,15,16,17,18,19]. However, the control of SETD8 levels through ubiquitin removal by deubiquitylating enzymes (DUBs) or ubiquitin hydrolases is less well characterized. Here, we identified USP29, a ubiquitin hydrolase with additional substrates in the DNA damage response, as a novel regulator of SETD8. USP29 controls SETD8 protein levels, and both proteins interact. Furthermore, USP29 deubiquitinates SETD8 in vivo and controls the DNA damage-induced monomethylation of H4K20 and focus formation of 53BP1. These results demonstrate that USP29 is a critical DUB for maintaining genome stability.

## 2. Materials and Methods

### 2.1. Cell Culture and Treatments

In this study, 293T, U2OS, and HeLa cells were grown in DMEM supplemented with 10% FBS and penicillin/streptomycin (Thermo Fisher Scientific, Waltham, MA, USA). Cells were synchronized at the G1/S transition by treatment with 2.5 mM thymidine (Merck-Millipore, Burlington, MA, USA) for 24 h. Cycloheximide (Merck-Millipore, Burlington, MA, USA) was used at 20 µg/mL, with MG132 (A.G. Scientific, San Diego, CA, USA) at 20 µM for 3–6 h or 5 µM for 16 h treatments, and cells were treated with ionizing radiation using a CellRad X-ray irradiator (Faxitron, Tucson, AZ, USA).

### 2.2. siRNA Oligos, Plasmids, and Transfection

siRNA oligonucleotides (20 µM, Merck-Millipore and Microsynth AG, Balgach, Switzerland) were transfected into cells using Lipofectamine RNAiMax (Thermo Fisher Scientific, Waltham, MA, USA). Sequences of oligonucleotides are presented below. siRNA oligonucleotides used to deplete USP29 are specified; otherwise, USP29#1 was used.
GFPCAAGCUGACCCUGAAGUUCdTdTLucCGUACGCGGAAUACUUCGAdTdTSETD8GUACGGAGCGCCAUGAAGUdTdTUSP29#1CCCAUCAAGUUUAGAGGAUdTdTUSP29#2GGAAUAUGCUGAAGGAAAUdTdTTRABIDAGAGGCUUCUUCAAUAAUAdTdTUSP18CCAGGGAGUUAUCAAGCAAdTdTUSP2CAGAUUGUGGUUACUGUUCUAdTdTUSP30CCAGAGUCCUGUUCGAUUUdTdTUSP33UCUCGACAGUGGCUUAAUUAAdTdTUSP51CCAUUUAGCUGUAGACCUUdTdT

Plasmid DNA was transfected into cells using the calcium phosphate transfection method [20]. The peGFPC1 USP29 wildtype (WT) and catalytic inactive (CI, C294S/H831N) plasmids were previously described [21]. pCMV SETD8-HA was kindly provided by Dr. Claus S. Sørensen (Biotech Research and Innovation Centre, University of Copenhagen, Copenhagen, Denmark), and PMT107 expressing His-ubiquitin was a gift from Dr. Dirk P. Bohmann (Rochester, New York, NY, USA).

### 2.3. Antibodies

Ku86 (C-20) was purchased from Santa Cruz Biotechnology, pSer139-H2AX (γH2AX, clone JWB301) was from Merck-Millipore, SETD8 (MA5-14804) and H4K20me1 (PA5-17027) were from Thermo Fisher Scientific, β-actin was from Genscript, and 53BP1 (Ab172580) was from Abcam.

Claspin [22], GFP [23], and USP29 [21] antibodies were published previously. The SETD8 antibody was generated by injecting a rabbit with a His-tagged antigen (amino acids 1–220) that was obtained through expression in bacteria and purification with a Ni-NTA resin (Qiagen, Hilden, Germany) following the manufacturer’s recommendations. Anti-HA (12CA5) was purified from a hybridoma cell line.

### 2.4. Immunoprecipitations, Pulldowns, and Western Blot

For immunoprecipitations, cells were lysed in EB150 lysis buffer (50 mM Hepes pH 7.5, 150 mM NaCl, 5 mM EDTA, 2 mM MgCl_2_, 0.5% NP40, 10% glycerol) for 20 min on ice. After centrifugation at 16,000 rpm for 20 min, extracts were incubated with antibody-coupled protein A-sepharose CL-4b (GE Healthcare, Chicago, IL, USA) for 2 h at 4 °C, followed by four washes with lysis buffer. The proteins were eluted with Laemmli buffer.

For pulldowns of His-tagged ubiquitinated proteins, cells were lysed in buffer A (8 M GuHCl, 10 mM Tris-HCl pH 8.0, 100 mM NaH_2_PO_4_, and 10 mM imidazole). After sonication, the extracts were incubated with Ni-NTA agarose beads (Genscript) for 2 h at RT, followed by three washes with buffer A, two with a 1:4 mix of buffer A:B (25 mM Tris-HCl pH 6.8, 20 mM imidazole), and finally two washes with buffer B. Sample buffer supplemented with 200 mM imidazole was added for elution.

Whole-cell extracts (WCEs) were prepared in urea/SDS buffer (6 M Urea, 1% SDS, 125 mM NaCl, 25 mM Tris pH 8). Lysates containing equal amounts of protein, measured by the BCA method (Thermo Fisher Scientific), were subjected to SDS-PAGE. Subsequently, proteins were transferred to nitrocellulose membranes (Amersham Protran Premium 0.45, Cytiva, Marlborough, MA, USA). Membranes were blocked with TBS-T + 5% nonfat milk, incubated with primary and HRP-conjugated secondary antibodies (Jackson Immunoresearch), and subsequently with SuperSignal West Pico Chemiluminescent Substrate (Thermo Fisher Scientific). Chemiluminescent images were obtained using the ImageQuant LAS 4000 mini and quantified using the Image Studio Lite Version 5.2.5 (LI-COR).

### 2.5. Immunofluorescence

Cells were fixed in 4% paraformaldehyde +0.2% Triton X-100 for 20 min and then permeabilized with methanol for 5 min at RT. For H4K20me1 staining, cells were pre-extracted (20 mM HEPES pH 8, 20 mM NaCl, 5 mM MgCl_2_, 0.5% NP40) for 5 min at 4 °C before fixation. Samples were blocked in PBS + 0.5% FBS, stained with antibodies as indicated and mounted with DAPI. Images were taken with the same exposure time for each fluorescent channel using a Zeiss Cell Observer fluorescence microscope equipped with a 20× NA 0.8 air immersion objective or a 63× NA 1.3 water immersion objective and ZEN imaging software. Analysis was performed using FIJI software (version 1.53p) [24]. The signal intensity of H4K20me1 in the nucleus was quantified as the H4K20me1 intensity minus the background signal. More than 100 cells were analyzed for each point.

### 2.6. Flow Cytometry

For cell cycle analysis, cells were fixed in 70% ethanol at 4 °C overnight. After fixation, cells were washed with PBS, and the DNA was stained with propidium iodide (PI). The samples were analyzed by flow cytometry using a MACSQuant Analyzer flow cytometer (Miltenyi, Bergisch Gladbach, Germany) and quantified using FlowLogic software version 8.6 (Inivai Technologies).

### 2.7. Clonogenic Survival

To determine cellular sensitivity to DNA damaging agents, HeLa cells were transfected with the indicated siRNA oligonucleotides. Then, 48 h later, 500 cells were seeded in six-well dishes and irradiated with the specified doses of IR the day after. Following 7–10 days in culture, cells were fixed and stained, and then colonies were counted. Triplicate cultures were scored for each treatment. The graph shows the relative survival as compared to the undamaged control, and the error bars present the standard error of four independent experiments.

### 2.8. Statistical Analysis and Reproducibility

The statistical differences between samples and experiments were analyzed in Prism software version 9.2.0.332 (GraphPad). For the one-way analysis of variance (ANOVA) followed by a Kruskal–Wallis multiple comparison test, a *p*-value < 0.05 was considered statistically significant (ns = not significant). Statistical significance was noted as follows: * *p* ≤ 0.05, ** *p* ≤ 0.01, *** *p* ≤ 0.001, and **** *p* ≤ 0.0001. Unless stated otherwise, representative experiments are shown, and the mean of the independent replicates ± standard deviation (SD) is depicted. Boxplots span the interquartile range, and the inside segments show the median value. Whiskers above and below indicate the maximum and minimum values, respectively. Additional details are listed in the individual figure legends.

When two variants were evaluated, the statistical significance of the difference was assessed by a two-way ANOVA, as in clonogenic survival analysis, or two-way repeated-measures ANOVA, as in cell cycle analysis, followed by Sidak’s multiple comparison test. Statistical significance was noted as follows: * *p* ≤ 0.03, ** *p* ≤ 0.002, *** *p* ≤ 0.0002, and **** *p* ≤ 0.00001.

## 3. Results

### 3.1. USP29 Controls SETD8 Protein Levels

**Figure 1:**
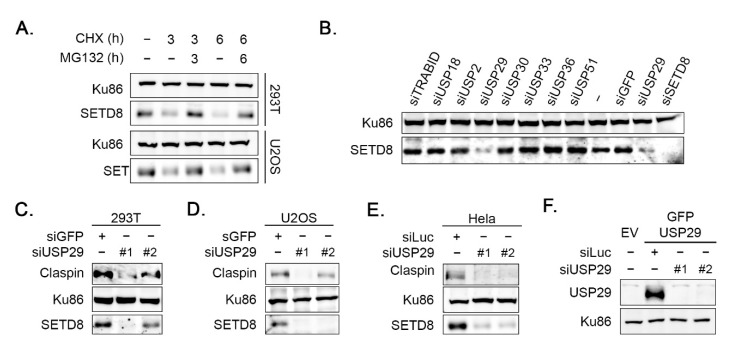
Depletion of USP29 affects SETD8 protein levels. (**A**) 293T cells and U2OS cells were treated with MG132 and/or CHX for 3 or 6 h. WCEs were analyzed by Western blot using the indicated antibodies. (**B**) Example of screening for ubiquitin hydrolases regulating SETD8 levels. U2OS cells were transfected with the specified siRNA oligonucleotides. Then, 48 h later, cells were lysed, and extracts were analyzed by Western blot with the indicated antibodies. (**C**) The 293T cells were transfected with GFP or the indicated USP29 siRNA oligonucleotides, lysed, and subsequently analyzed by Western blot using the indicated antibodies. (**D**) As in (**C**), but in U2OS cells. (**E**) HeLa cells were transfected with Luciferase (Luc) or the indicated USP29 siRNA oligonucleotides. WCEs were analyzed by Western blot using the indicated antibodies. (**F**) U2OS cells were transfected with an empty vector (EV) or GFP-USP29 and thereafter transfected with Luc or USP29 siRNA oligonucleotides. Extracts were analyzed by Western blot analysis with the indicated antibodies.

Recent studies have shown that SETD8 protein levels are controlled by ubiquitin-mediated proteasomal degradation [25]. This was confirmed by our experiments in U2OS and 293T cells, demonstrating that the decreased SETD8 protein levels upon treatment with cycloheximide (CHX) could be rescued by incubating the cells with proteasome inhibitor MG132 (Figure 1A). Although several E3 ubiquitin ligases were described to be involved in the regulation of SETD8 protein stability, at the start of this study, nothing was known about how deubiquitination of SETD8 is controlled [10,15,16,17,18,19]. To identify novel regulators related with the (de)ubiquitination of SETD8, we performed a screening in U2OS cells using a library of siRNA oligonucleotides for the majority of human ubiquitin hydrolases, using SETD8 levels measured by Western blotting as readout (Figure 1B). Several candidate DUBs that affect the level of SETD8 were identified, among which USP29 was the most obvious one, as depletion of USP29 resulted in a decrease in SETD8 protein levels (Figure 1B and data not shown). Using an additional siRNA oligonucleotide to downregulate USP29 had the same effect, albeit with different efficiency, in U2OS, 293T, and HeLa cells (Figure 1C–E). These results exclude a possible off-target effect by the siRNA oligonucleotides and show that USP29 controls SETD8 protein levels. In these experiments, DNA damage mediator protein Claspin, previously described as a USP29 substrate, was used as a positive control [21] (Figure 1C–E). Additionally, although we were unable to detect endogenous USP29 due to the lack of a specific antibody, transfecting cells overexpressing USP29 with siRNA oligonucleotides resulted in downregulation of this version of the protein, demonstrating the knockdown efficiency with the used oligonucleotides (Figure 1F).

### 3.2. USP29 Regulates SETD8 Levels in a Cell-Cycle-Independent Manner

**Figure 2:**
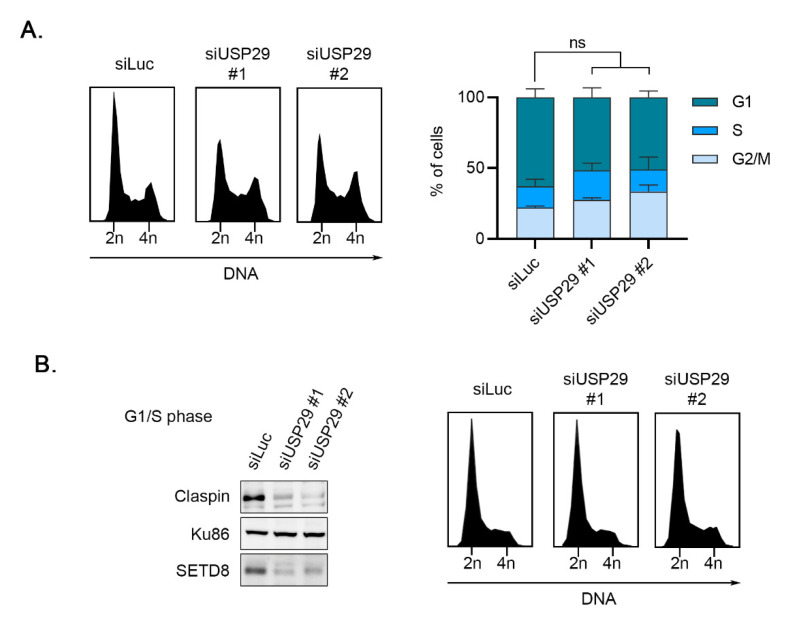
USP29 controls SETD8 independently of the cell cycle. (**A**) U2OS cells were transfected with Luc or the indicated USP29 siRNA oligonucleotides and analyzed for PI by flow cytometry (left panel). Quantification shows the percentage of cells in G1, S, and G2/M phases of three independent experiments (right panel). (**B**) U2OS cells depleted as in (**A**) were synchronized by thymidine block and analyzed by Western blot analysis with the indicated antibodies (**left** panel) or by flow cytometry for PI (**right** panel).

SETD8 protein levels were described to fluctuate during the cell cycle, with degradation at the G1/S transition, resulting in very low levels during the S phase, which rise again in G2 phase [10,15,16,17]. The effect of USP29 depletion on SETD8 levels could, therefore, be explained by a possible indirect effect on the cell cycle. Analyzing cell cycle profiles upon depletion of USP29 by flow cytometry showed that USP29 depletion did not cause major changes in cell cycle progression (Figure 2A). In addition, depleting USP29 in cells synchronized at the G1/S transition using thymidine treatment led to a similar decrease in SETD8 protein levels as in asynchronous cells (Figure 2B), thereby ruling out the possibility that lower SETD8 levels are caused by an indirect effect on cell cycle progression upon knockdown of USP29.

### 3.3. SETD8 and USP29 Interact In Vivo

**Figure 3:**
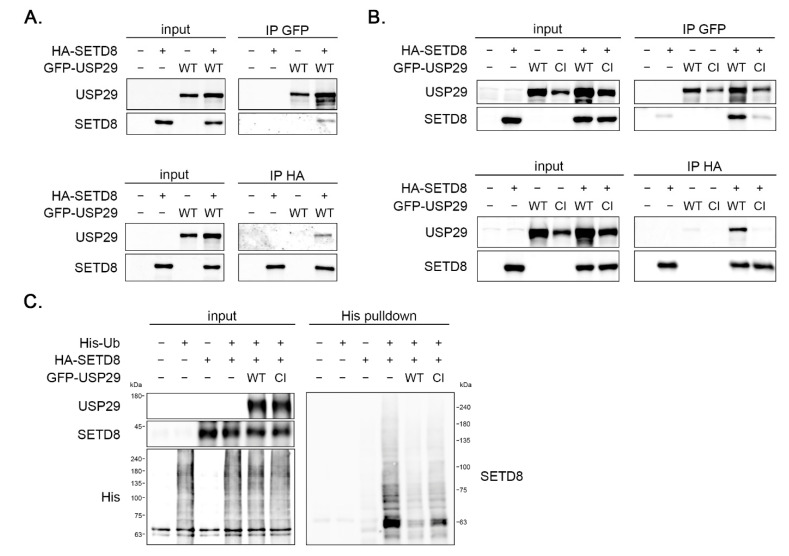
USP29 interacts with and deubiquitinates SETD8 in vivo. (**A**) The 293T cells were transfected with HA-SETD8 and/or GFP-USP29 wildtype (WT) expression vectors and lysed. GFP (**upper** panel) or HA (**lower** panel) immunoprecipitations were carried out and analyzed by Western blot with the indicated antibodies. (**B**) The 293T cells were transfected with HA-SETD8 and WT or catalytic inactive (CI) versions of GFP-USP29. GFP (**upper** panel) or HA (**lower** panel) immunoprecipitations were carried out and analyzed by Western blot with the indicated antibodies. (**C**) The 293T cells were transfected with HA-SETD8, His-Ub, and GFP-USP29 WT or CI. Cells were incubated with MG132 for 16 h before lysis. Then, a pulldown was performed with Ni-NTA beads followed by Western blot analysis using the indicated antibodies.

The explore if the effect of USP29 on SETD8 might be direct, the interaction between these proteins was tested. Tagged versions of USP29 and SETD8 were overexpressed in 293T cells, after which the proteins were immunoprecipitated and analyzed by Western blot. When immunoprecipitating GFP-USP29, SETD8 was pulled down (Figure 3A, upper panel). Vice versa, USP29 was found in immunoprecipitations of HA-SETD8 (Figure 3A, lower panel). These data demonstrate that SETD8 and USP29 interact in vivo. To test if the catalytic activity of USP29 was required for this interaction, HA-SETD8 was expressed, together with wildtype (WT) or a catalytic inactive version (CI) of GFP-USP29. SETD8 co-immunoprecipitated with GFP-USP29 WT, but to a lesser extent with GFP-USP29 CI (Figure 3B, upper panel). Likewise, the WT, but not the CI version of USP29 was detected after immunoprecipitating HA-SETD8 (Figure 3B, lower panel). Together these data show that SETD8 and the USP29 bind to each other in vivo, and that this interaction depends on the ubiquitin hydrolase activity of USP29. This suggests that the effect of USP29 on SETD8 protein levels is direct, most likely by controlling the ubiquitination status of SETD8.

### 3.4. USP29 Deubiquitinates SETD8 In Vivo

To study if USP29 could control SETD8 protein levels via direct deubiquitination, in vivo ubiquitination assays were performed. The 293T cells were transfected with His-Ubiquitin (His-Ub), HA-SETD8, and GFP- USP29 WT or CI and incubated with MG132 to prevent degradation of polyubiquitinated proteins. Subsequently, ubiquitin-conjugated proteins were pulled down and analyzed by Western blot (Figure 3C). Abundant SETD8 polyubiquitination was detected, which was significantly decreased upon co-expression of USP29 WT, but to a lesser extent by expression of the hydrolase mutant version of USP29 (Figure 3C). These data demonstrate that USP29 directly stabilizes SETD8 via deubiquitination.

### 3.5. Accumulation of 53BP1 into Nuclear Foci upon IR Is Regulated by USP29

**Figure 4:**
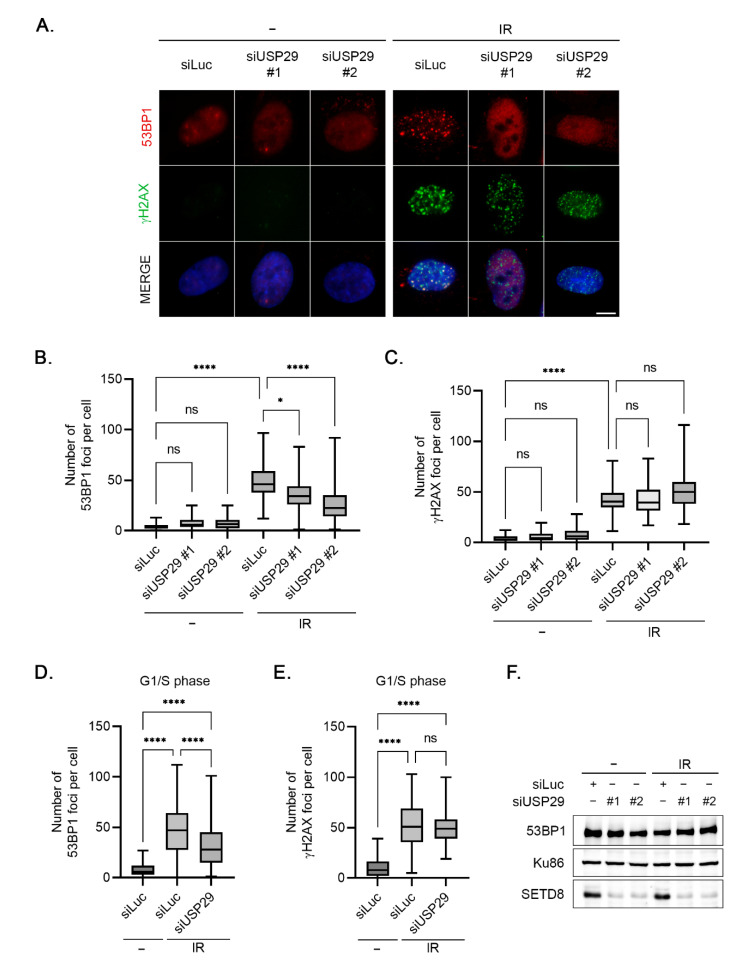
USP29 is required for 53BP1 focus formation upon IR. (**A**) U2OS cells were transfected with Luc or the indicated USP29 siRNA oligonucleotides. Then, 48 h later, the cells were left untreated or treated with IR (4 Gy) and fixed after 1 h; focus formation by 53BP1 and γH2AX was analyzed by IF. Representative images are shown. Scale bar, 10 µm. (**B**) Quantification of the number of 53BP1 foci per cell of two independent experiments, from (**A**). (**C**) As in (**B**), but for focus formation of γH2AX. (**D**) U2OS cells were depleted of Luc or USP29 by siRNA, after which the cells were arrested at G1/S transition by thymidine block and treated with IR (4 Gy, 1 h); focus formation of 53BP1 was analyzed by IF. Quantification of number of 53BP1 foci per cell of one representative experiment. (**E**) As in (**D**), but for focus formation by γH2AX. (**F**) U2OS cells were transfected and treated as in (**A**). Cells were lysed, and extracts were analyzed by Western blot with the indicated antibodies.

SETD8 is a histone methyltransferase, with histone H4K20 as one of its critical substrates [10]. Recruitment of 53BP1, a mediator protein in the DNA damage response, to sites of DNA lesions critically depends on H4K20 methylation, as 53BP1 binds this mark via its tandem Tudor domains [26,27]. Interestingly, recent data showed that depletion of SETD8 abrogates the accumulation of 53BP1 to sites of DNA lesions [12]. To study the functional consequences of regulation of SETD8 by USP29, we analyzed the effect of USP29 depletion on the DNA damage-induced focus formation of 53BP1 by immunofluorescence (IF) (Figure 4A). In control conditions, treating the cells with ionizing radiation (IR) triggered the accumulation of 53BP1 into nuclear foci, whereas the increase in 53BP1 nuclear foci in response to IR was prevented in cells depleted of USP29 (Figure 4A,B). In contrast and as expected, focus formation of γH2AX, an earlier event in the DNA damage response and independent of H4K20 methylation, was unaffected by downregulation of USP29 (Figure 4A,C). These data demonstrate that the absence of USP29 inhibits DNA damage-induced recruitment of 53BP1 to sites of DNA lesions, likely via affecting H4K20 methylation by modulating the protein levels of SETD8. To rule out any nonspecific effect of cell cycle progression, the same experiment was performed in cells synchronized at the G1/S transition by treatment with thymidine. In these cells, depletion of USP29 also led to a lower number of IR-induced nuclear foci of 53BP1 as compared to control knockdown cells, whereas the absence of USP29 left the γH2AX focus formation after IR unaffected (Figure 4D–E). Importantly, 53BP1 protein levels remained unchanged after USP29 depletion (Figure 4F), indicating that USP29 does not control 53BP1 levels but instead directly regulates the accumulation of 53BP1 into nuclear foci upon IR.

### 3.6. USP29 Controls IR-Induced H4K20 Monomethylation and Cell Survival

**Figure 5:**
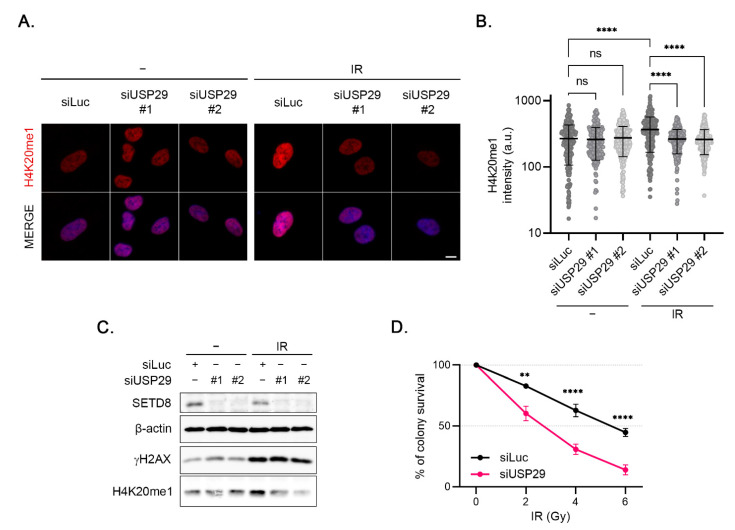
USP29 controls IR-induced H4K20 monomethylation and cell survival. (**A**) U2OS cells were transfected with Luc or the indicated USP29 siRNA oligonucleotides, treated with IR (4 Gy, 1 h), and analyzed for nuclear H4K20me1 intensity by IF. Representative images are shown. Scale bar, 10 µm. (**B**) Quantification of nuclear H4K20me1 intensity per cell of two independent experiments. (**C**) U2OS cells were transfected and treated as in (**A**). Cells were lysed and extracts were analyzed by Western blot with the indicated antibodies. (**D**) Colony formation of HeLa cells, depleted of Luc or USP29 and treated with the indicated doses of IR. Shown is the relative survival as compared to the undamaged control. Error bars represent the SEM of four individual experiments.

As mentioned above, 53BP1 focus formation depends on SETD8-dependent methylation of H4K20. To explain the defect in 53BP1 focus formation upon depletion of USP29, we examined the effect of USP29 knockdown on the monomethylation of H4K20 by IF (Figure 5A). USP29 depletion did not affect H4K20me1 levels in undamaged conditions (Figure 5A,B). Interestingly however, treating the cells with IR led to a significant increase in H4K20me1 levels, which was inhibited by the downregulation of USP29 (Figure 5A,B). Western blot analysis confirmed these results. Whereas USP29 knockdown did not affect H4K20 monomethylation in undamaged cells, depletion of this DUB prevented the IR-induced increase in H4K20 monomethylation (Figure 5C). These data strongly suggest that USP29 controls 53BP1 focus formation via regulating SETD8-mediated H4K20 monomethylation.

Lastly, colony formation assays were performed to examine the long-term effect of USP29 absence in conditions of DNA damage. Depletion of USP29 resulted in an increased sensitivity to IR in HeLa cells (Figure 5D). Together, these data demonstrate that USP29 is critical for the DNA damage response and cell survival, likely via its role in controlling protein levels of SETD8.

## 4. Discussion

This study investigated novel regulatory factors of the histone methyl transferase SETD8, which acts as an epigenetic writer through monomethylation of histone H4K20 and is, thus, critical for a diverse range of pathways in the cell [3,5,28]. SETD8 protein levels were shown to be regulated by ubiquitin-mediated proteasomal degradation during the cell cycle and after DNA damage, and several E3 ubiquitin ligases have been implicated in the control of SETD8 protein stability, including SCF^Skp2^, APC^Cdh^1, CRL4^Cdt2^, and SCF^βTrCP^ [10,15,16,17,18,19]. Here, we aimed to characterize novel regulators of SETD8 among human ubiquitin hydrolases by performing an siRNA screening. In this screening, USP29 was identified a regulator of SETD8 as downregulation of USP29 led to decreased SETD8 protein levels. SETD8 is not the first USP29 substrate that is involved in the responses controlling genomic stability. USP29 was shown to bind and deubiquitinate tumor suppressor p53 in vivo, thereby stabilizing the protein [29]. Others also reported elevated p53 and MDM2 levels upon overexpression of USP29, along with an increase in histone H2A monoubiquitination, a critical modification in the response to DNA double-strand breaks (DSBs), in the same circumstances [30]. However, as the overexpression of USP29 reversed the ubiquitination of several other factors, including PCNA, a processivity factor for DNA polymerase, it remains to be demonstrated if these are bona fide USP29 substrates or if this is a nonselective effect due to the overexpression of USP29 [30]. Furthermore, USP29 was shown to stabilize Claspin, a critical mediator in the DSB response, via interaction and direct deubiquitination [21]. With several substrates among proteins functioning in the DNA damage response, USP29 can be considered as one of the central ubiquitin hydrolases contributing to the maintenance of genome integrity.

Our results show that the decreased levels of SETD8 in cells depleted of USP29 do not have an indirect effect on cell cycle progression and, moreover, demonstrate an interaction between USP29 and SETD8 in vivo. Interestingly, a catalytic inactive version of USP29 did not coimmunoprecipitate with SETD8, indicating that the ubiquitin hydrolase activity is required for the interaction of SETD8 with USP29. The SETD8–USP29 interaction suggests that USP29 might control SETD8 by directly removing polyubiquitin chains from SETD8, thereby stabilizing the protein. Indeed, overexpression of wildtype USP29 diminished polyubiquitination of SETD8 in vivo, whereas the polyubiquitinated SETD8 was less affected by a co-expression of a catalytic inactive version of the ubiquitin hydrolase.

We next demonstrated that the depletion of USP29 inhibited the accumulation of 53BP1 into foci after treating the cells with IR. These data seem in contrast to those of others, which demonstrated that overexpression of wildtype, but not catalytic inactive USP29 prevented IR-induced 53BP1 focus formation. It was hypothesized that USP29 antagonizes RNF168-mediated ubiquitination of H2A [30]. However, as overexpression of other DUBs, previously described to control H2A via direct deubiquitination, did not inhibit 53BP1 focus formation, overexpression of USP29 was thought to have nonspecific effects [30,31]. Indeed, our data are in accordance with studies describing that depletion of SETD8 inhibited accumulation of 53BP1 to sites of laser-induced damage or IR-induced focus formation of 53BP1 and DSB repair by NHEJ, an effect explained by lower H4K20 methylation, important for the localization of 53BP1 to sites of damage, in conditions of depletion of SETD8 [10,12].

Moreover, we showed that DNA damage results in an increase in overall monomethylation of H4K20. These data are in agreement with the published accumulation of H4K20 methylation at sites of local damage [11,12,13]. Importantly, this IR-induced increase in H4K20 monomethylation was prevented by knockdown of USP29. These data strongly suggest that USP29 is critical for 53BP1 focus formation by regulating H4K20 monomethylation via controlling SETD8 protein levels. This event is critical for maintaining genome stability, as demonstrated by increased sensitivity to IR in cells downregulated for USP29. However, as USP29 has additional substrates among proteins functioning in the DNA damage response, as well as possibly other so far unidentified substrates, the observed sensitivity to IR upon depletion of USP29 might be caused by its effect on SETD8 levels, in addition to other effects.

While our work was ongoing, another group reported regulation of SETD8 protein levels by USP17. This ubiquitin hydrolase and SETD8 interact, and USP17 deubiquitinates polyubiquitinated SETD8 in vivo and in vitro. Knockdown of USP17 affected H4K20 methylation and led to elevated p21 levels, thereby inducing a G1 arrest and cellular senescence [32]. Control of SETD8 by USP17 was furthermore suggested to activate lipid biosynthesis and to contribute to clear-cell renal cell carcinoma development via the SREBP1 pathway [33]. These data underscore the versatile role of SETD8 in contributing to the maintenance of genome integrity. Interestingly, our screening resulted in additional candidate DUBs that might control SETD8 protein levels. The implications of these ubiquitin hydrolases on regulating SETD8 and the DNA damage response will be studied in further detail in the future.
